# Post-extubation stridor after prolonged intubation in the pediatric intensive care unit (PICU): a prospective observational cohort study

**DOI:** 10.1007/s00405-020-05877-0

**Published:** 2020-03-04

**Authors:** L. L. Veder, K. F. M. Joosten, K. Schlink, M. K. Timmerman, L. J. Hoeve, M. P. van der Schroeff, B. Pullens

**Affiliations:** 1 Department of Otorhinolaryngology and Head and Neck Surgery, Erasmus Medical Center, Sophia Children’s Hospital, Rotterdam, The Netherlands; 2 Department of Pediatric Intensive Care, Erasmus Medical Center, Sophia Children’s Hospital, Rotterdam, The Netherlands; 3 Department of Otorhinolaryngology, Erasmus Medical Center, Sophia Children’s Hospital, Room SP-1421a, Rotterdam, The Netherlands

**Keywords:** Prolonged intubation, Laryngeal damage, Pediatric airway, Stridor, Complications, Laryngotracheal stenosis, Subglottic stenosis, Endotracheal tube

## Abstract

**Purpose:**

Prolonged endotracheal intubation may lead to laryngeal damage, with stridor being the most relevant clinical symptom. Our objective was to determine the incidence of post-extubation stridor and their clinical consequences in children within a tertiary referral center and to identify contributing factors.

**Methods:**

150 children, aged 0–16 years, intubated for more than 24 h were prospectively enrolled until discharge of the hospital. Potential relevant factors, thought to mediate the risk of laryngeal damage, were recorded and analyzed.

**Results:**

The median duration of intubation was 4 days, ranging from 1 to 31 days. Stridor following extubation occurred in 28 patients (18.7%); 3 of them required reintubation due to respiratory distress and in 1 child stridor persisted for which a surgical intervention was necessary. In multivariate analyses, we found the following independent predictors of stridor: intubation on the scene, the use of cuffed tubes and lower age.

**Conclusion:**

Despite a high incidence for post-extubation stridor, only few children need reintubation or surgical intervention as a result of post-extubation lesions. Intubation on the scene, the use of cuffed tubes and young age are associated with a significant increased risk of post-extubation stridor. Awareness of these factors gives the possibility to anticipate on the situation and to minimize laryngeal injury and its possible future consequences.

## Introduction

Prolonged endotracheal intubation may lead to laryngeal damage. Pressure from the tube is thought to cause ischemia and consequently erosion and ulceration of the laryngeal mucosa; finally, resulting in the formation of scar tissue. The lesions occur at the points of greatest pressure, involving the posterior glottis at the medial aspect of the arytenoid cartilages, at the superior part of the cricoid lamina and in the cricoid itself [1, 2].

Previous studies have shown that the majority of patients show some form of laryngeal injury after extubation, varying from mild edema to vocal fold immobility. However, most of these injuries go unnoticed and heal spontaneously with no or minimal consequences [3–7]. Infrequently, post-extubation laryngeal lesions lead to a life-threatening laryngotracheal stenosis, which presents with typical signs of upper airway obstruction like chest retractions, dyspnea, and inspiratory stridor.

The development of post-extubation laryngeal injuries is thought to be a multifactorial phenomenon. Several significant associated factors leading to laryngeal injury and laryngotracheal stenosis have been identified like duration of intubation, multiple intubations, and infection [5, 8–13]. The factors age, gender, low gestational age, low birth weight, (congenital) narrow larynx, gastro-esophageal reflux, traumatic intubation, and inappropriate tube size are also considered to contribute to the development of laryngeal intubation injury. However, literature is ambiguous with differing results [3, 8–12, 14–18]. Also, the use of a cuffed tube in children under the age of 8 years has been under debate due to the concerns for laryngeal injury leading to laryngotracheal stenosis. However, since the development of “high volume low pressure” cuffed endotracheal tubes, and the introduction of the ‘Microcuff^®^’ pediatric endotracheal tube, multiple studies have shown that the cuffed tube is safe for use in children under the age of eight. Even the use of a ‘Microcuff^®^’ endotracheal tube in neonates weighing less than three kilograms may be safe [16, 18–21].

Insight on post-extubation stridor with its clinical consequences and identification of predisposing factors associated with post-extubation stridor is important in order to early diagnose post-extubation lesions and to enable early therapies, making the therapies more effective.

Our primary objectives were to determine the incidence of post-extubation stridor in children after prolonged intubation as a proxy for laryngeal damage, to investigate its clinical consequences and to investigate associated factors that contribute to post-extubation stridor.

## Methods

### Ethical considerations

The Clinical Research Ethics Committee at our hospital approved this study and informed consent was not mandated. No additional investigations or interventions were performed in the interests of this study. The medical decision making was left to discretion of the attending physician.

### Participants

From June 2010 until June 2011, all children admitted to the Pediatric Intensive Care Unit (PICU) in the Erasmus Medical Center-Sophia Children’s Hospital, intubated for more than 24 h, were prospectively enrolled. Children were followed during admission until the day of discharge from the hospital. Exclusion criteria for the study were known congenital or acquired anomalies of the larynx or trachea, stridor prior to intubation, the need for a tracheal cannula due to prolonged intubation, death before extubation or death within 24 h after extubation, extubation due to withdrawal of treatment, and extubation outside the Sophia Children’s Hospital.

### Data

Data were collected from the Patient Data Management System (PDMS), electronic patient system (Elpado and Hix) and medical charts at the PICU by personnel who were not involved in medical decision making. Stridor, age, gender, weight, indication for intubation, medical history, location of intubation, physician who intubated, tube characteristics, air leak, current infection, duration of intubation, need for tube change and use of inotropics, steroids, or antibiotics during intubation were analyzed. Stridor was reported clinically by the attending physician. We did not collect data regarding reflux.

The correct size of the endotracheal tube was computed using the formula: (age in years)/4 + 4 = endotracheal tube internal diameter (mm). If a tube was selected that was a half-size bigger than predicted by the formula this was considered incorrect. Airleak was computed only for uncuffed tubes by the formula: Air leak = (Ins TV − Exp TV)/Ins TV * 100%. The term ‘tube change’ was used for those who required a tube change after auto-extubation, tube block or because of excessive air leak. Respiratory infections had to be confirmed by a positive sputum culture. Any treatment for post-extubation respiratory distress was noted. This included nebulizing with steroids or epinephrine, respiratory support with high flow oxygen (Optiflow^®^) or a nasopharyngeal tube, diagnostic endoscopy, and surgical interventions.

In patients who were intubated more than once during the study period, every endotracheal intubation was seen as a separate event, except when there was less than 24 h between two episodes of intubation. In this way, some patients have multiple intubation records. For the analysis, we only used the first record or in case of stridor, we used the record where the patient developed stridor.

### Statistical analysis

Patient baseline characteristics were described in median with range or in number and percentage. Univariate risk factor analysis was followed by multivariate analysis to define which of the main variables were independently associated with the outcome of post-extubation stridor. In the multivariate analysis, we included location of intubation, the use of cuffed tubes, age, intubation length, the use of steroids prior to extubation, the use of correct tube sizes, presence of infections, and underlying syndromes. An odds ratio with 95% confidence interval, and *p* value was established by binary logistic regression. We also stratified for the age groups 0–1 year old, 1–8 years old and 8–16 years old. A *p* < 0.05 was considered statistically significant. All statistical analyses were performed using SPSS version 25 (IBM, Chicago, USA).

## Results

**Fig. 1:**
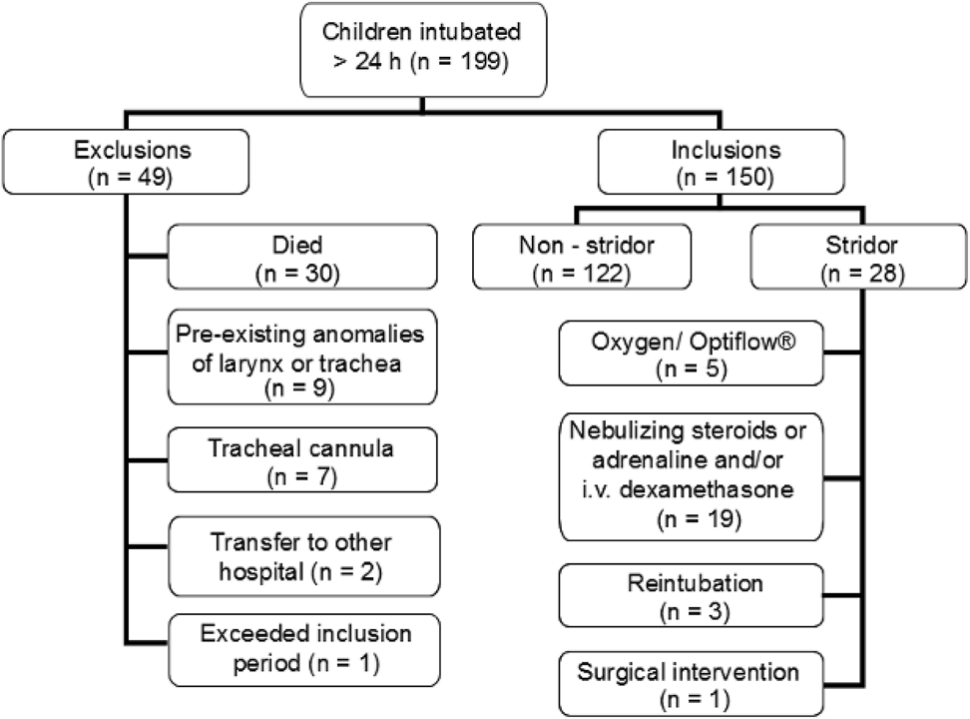
Flowchart inclusion and follow-up patients

During the inclusion period, 199 patients were admitted to the PICU of Sophia Children’s Hospital and were intubated for more than 24 h. Forty-nine patients were excluded from the study. Accordingly, the study population consisted of 150 patients aged from 0 to 16 years. Stridor following extubation occurred in 28 patients (18.7%). In 5 patients with stridor (17.9%), it resolved without any additional treatment besides oxygen therapy or Optiflow^®^. Treatment with nebulizing steroids or adrenaline, or intravenous dexamethasone was sufficient to resolve stridor in 19 patients (67.9%). Three patients (10.7%) required reintubation due to respiratory distress. One patient (3.6%) underwent endoscopic dilatation due to an acquired subglottic stenosis; see Fig. 1.

One of the children that needed reintubation was a 2-year-old boy, primarily intubated for an elective dental operation. He underwent endoscopy in general anesthesia revealing bilateral subglottic mucosal lesions due to the tube. The subsequent extubation was done 2 days later with administering dexamethasone beforehand. Afterwards, there were no respiratory problems. The other two patients with stridor that had to be reintubated, also appeared to have underlying cardiac and pulmonary problems; for which, they had to be treated first. Afterwards one of these children was extubated without any problems. The other child received dexamethasone before and after extubation and also needed nebulizing with adrenaline. She too was extubated without further incidents. The one child that required endoscopic dilatation of an acquired subglottic stenosis was a 1-year old boy who developed stridor 2 weeks after extubation. One month after the intervention, a re-dilatation was successfully performed once again because of restenosis. No further intervention was necessary afterwards. None of the patients with stridor required a tracheal cannula.

Baseline characteristics are shown in Table 1. Intubation on the scene was done by ambulance staff or the Mobile Medical Team. No bedside fiberoptic laryngoscopy was performed.

**Table 1:** Baseline characteristics

Variables	Total (*n* = 150)	Stridor (*n* = 28)	Non-stridor (*n* = 122)	*p*
Duration of intubation (days)	4.0 (1–31)^a^	3.5 (1–25)^a^	4.0 (1–31)^a^	ns
Age (months)	1.0 (0–201)^a^	9.0 (0–186)^a^	1.0 (0–201)^a^	ns
Boy	89 (59.3%)	15 (53.6%)	74 (60.7%)	ns
Weight at intubation (kg)	4.2 (1.7–90)^a^	8.0 (2.0–90)^a^	4.0 (1.7–65)^a^	ns
Indication for intubation				ns
Respiratory insufficiency	68 (45.3%)	10 (35.7%)	58 (47.5%)	
Surgical intervention	61 (40.7%)	11 (39.3%)	50 (41.0%)	
Trauma	6 (4.0%)	3 (10.7%)	3 (2.5%)	
Cardiac instability	5 (3.3%)	1 (3.6%)	4 (3.3%)	
Neurological	5 (3.3%)	1 (3.6%)	4 (3.3%)	
Others	5 (3.3%)	2 (7.1%)	3 (2.5%)	
Intubation in				0.03
Sophia children’s hospital	99 (66.0%)	15 (53.6%)	84 (68.9%)	
Another hospital	38 (25.3%)	7 (25.0%)	31 (25.4%)	
At the scene	13 (8.7%)	6 (21.4%)	7 (5.7%)	
Intubated by				ns
Anesthetist	71 (47.3%)	11 (39.3%)	60 (49.2%)	
Intensivist	28 (18.7%)	4 (14.3%)	24 (19.7%)	
‘Another physician’	51 (34.0%)	13 (46.4%)	38 (31.1%)	
Nasal intubation	99 (66.0%)	19 (67.9%)	80 (65.6%)	ns
Oral intubation	51 (34.0%)	9 (32.1%)	42 (34.4%)	
Correct tube size	148 (98.7%)	27 (96.4%)	121(99.2%)	ns
Cuffed tube	41 (27.3%)	11 (39.3%)	30 (24.6%)	ns
Air leak ≤ 10% (uncuffed tubes)	43 (38.5%)	5 (29.4%)	37 (40.2%)	ns
Air leak > 10% (uncuffed tubes)	60 (55.0%)	11 (64.7%)	49 (53.3%)	
Missing	7 (6.4%)	1 (5.9%)	6 (6.5%)	
Intubated > 7 days	41 (27.3%)	6 (21.4%)	35 (28.7%)	ns
Intubated ≤ 7 days	109 (72.7%)	22 (78.6%)	87 (71.3%)	
No tube change	44 (29.3%)	9 (32.1%)	35 (28.7%)	ns
No syndrome	138 (92.0%)	25 (89.3%)	113 (92.6%)	ns
Down syndrome	5 (3.3%)	2 (7.1%)	3 (2.5%)	
Another syndrome	7 (4.7%)	1 (3.6%)	6 (4.9%)	
No infection	72 (48.0%)	13 (46.4%)	59 (48.4%)	ns
Respiratory infection	49 (32.7%)	9 (32.1%)	40 (32.8%)	
Another infection	28 (18.7%)	6 (21.4%)	22 (18.0%)	
Missing	1 (0.7%)	0	1 (0.8%)	
No shock	93 (62.0%)	15 (53.6%)	78 (63.9%)	ns
Antibiotics used	107 (71.3%)	19 (67.9%)	88 (72.1%)	ns
Steroids prior to extubation	25 (16.7%)	8 (28.6%)	17 (13.9%)	ns

*ns* not significant

^a^Data presented in median (range) or number (percentage)

Multivariate analysis is shown in Table 2. Intubation on the scene, the use of cuffed tubes, and young age were found to be significantly associated with stridor in the entire group. After stratification for the age groups, 0–1 year old, 1–8 years old, and 8–16 years old, intubation on the scene was only found to be significantly associated with stridor in children between 1 and 8 years old. The use of cuffed tubes was only found to be significantly associated with stridor in the age group 0–1 year old. Intubation for more than 1 week and the use of steroids before extubation showed a trend towards significance; 25 children had steroids prior to extubation; of which, 8 (32%) developed stridor.

**Table 2:** Multivariate analysis on post-extubation stridor

Variables	OR (95% CI)	*p*
Intubation at the scene	5.59 (1.39–22.48)	0.02
Cuffed tube	4.69 (1.23–17.91)	0.02
Age (months)	0.99 (0.97–1.00)	0.04
Intubation > 7 days	0.27 (0.07–1.00)	0.05
Steroids prior to extubation	3.08 (0.97–9.75)	0.06
Correct tube size	3.68 (0.16–87.66)	0.42
Infection	1.11 (0.43–2.89)	0.83
Syndrome	2.98 (0.58–15.25)	0.19

Twenty-one patients < 8 years old were intubated with a cuffed tube, 3 (14.3%) of them were < 1 year. In none of the children, a ‘Microcuff^®^’ endotracheal tube was used; during the inclusion period, other endotracheal cuffed tubes were used.

## Discussion

This prospective and well-documented study reveals ‘intubation on the scene’, ‘the use of cuffed tubes’, and ‘young age’ as contributing factors in post-extubation stridor in relation with the routine care at the PICU in our tertiary referral center. In almost all patients, stridor resolved with or without nebulizing treatment, 1 patient needed reintubation due to bilateral subglottic mucosal lesions. Subsequently, 1 patient with stridor developing 2 weeks after extubation required an endoscopy and surgical intervention due to acquired subglottic stenosis.

In our study, the incidence of stridor was 18.7%, whereas in the previous literature an incidence varying from 2 to 42% was reported [11, 21–23]. Thus, there is a high incidence of post-extubation stridor, which generally reacted on conservative treatment, and the incidence of a subglottic stenosis in this study was low. Schweiger et al. [24] previously showed that persisting stridor for over 72 h after extubation or an onset of stridor after 72 h post-extubation is highly specific for a laryngotracheal stenosis. This is in agreement with our patient with a subglottic stenosis who developed stridor after 2 weeks.

An associated factor we have found for post-extubation stridor is the site of intubation. Patients who had been intubated outside the hospital were 5 times more likely to develop stridor afterwards, compared to patients intubated in a hospital. This is possibly because emergency intubations on the streets are more traumatic due to unideal circumstances with physicians less experienced in intubating small children. Ehrlich et al. [25] noted that the success rate of an endotracheal intubation differs significantly by site, with the majority of complications occurring in the field or at a referring hospital. The relative rarity of intubating children in the field and the difficult anatomy of a child with a small and anterior placed airway are contributing to the lower success rate of intubation in the field.

The use of cuffed tubes in children, aged between 0 and 1 year old, was significantly associated with developing stridor after extubation, whereas no association was found for children aged between 1 and 8 years old and children aged between 8 and 16 years old. The use of cuffed tubes in small children has been under debate. Traditional pediatric airway management advised against the use of cuffed endotracheal tubes in children under the age of eight. However, the recent literature showed when using an “high volume low pressure” cuffed endotracheal tube in small children, no evidence for differences between cuffed and uncuffed tubes for outcomes like the need to treat post-extubation stridor was found. Moreover, the use of a cuffed tube decreases the need for tube exchange [11, 16, 19–21, 23, 26, 27]. In our study no “high volume low pressure” cuffed endotracheal tubes were used, only other ‘old-fashioned’ conventional endotracheal cuffed tubes were used. Our results support the recommendation not to use latter tubes in children aged between 0 and 1 year old.

During this study, cuffed endotracheal tubes were not routinely used in children under 8 years, although 21 (14%) children under an age of 8 years received a cuffed tube, due to the need for high inspiratory pressures with mechanical ventilation. Information regarding handling of the cuffed tube, like pressure of the cuff, is lacking.

Corticosteroids are believed to reduce the inflammatory response and to decrease laryngeal edema and; therefore, to lower the incidence of post-extubation stridor. The use of steroids prior to extubation in adults has been confirmed to be useful. Studies in children have reported a trend towards benefit, but have not proven to be effective [28, 29]. In our hospital, steroids are not given routinely. In patients where difficulties at extubation are likely, like patients with Down syndrome, craniofacial syndromes or patients with laryngeal and/ or tracheal anomalies, steroids (dexamethasone 0.5 mg/kg) are given 6 h and 30 min prior to extubation, which was the case in 25 (16.7%) patients. In our multivariate analysis, the use of steroids before extubation showed a trend (*p* = 0.06) towards significance in patients who developed stridor. We think this inverse association underlines the need for proper selection of the group of children with potential complications during extubation. By reducing mucosal swelling and airway obstruction, corticosteroids might prevent poorer outcomes.

Although literature remains controversial, duration of intubation is considered to play a major role in the post-extubation outcome of the patient [3, 11], and our study also showed a trend towards significance in developing stridor after intubation for more than a week. One could consider whether children intubated for more than a week would benefit from the use of steroids before extubation.

Also, based on the previous studies [5, 8, 22], one would have predicted that underlying syndromes and the presence of infections would have increased the incidence of stridor. Yet, in our study group, we did not observe an increase in the incidence of stridor after correcting for these variables, but the sample size of these factors might have been too low.

A limitation is the lacking correlation between stridor and laryngeal injury. Awake flexible fiberoptic laryngoscopy can be used to identify glottic and supraglottic laryngeal pathology, but only endoscopy in general anesthesia can reliably detect all airway injuries caused by prolonged intubation. As this is an observational study, we were not able to routinely perform endoscopic evaluation after extubation in all children. As shown in our single patient with a subglottic stenosis, stridor might develop weeks or months after extubation when silent ulcerations of the mucosa retract to a stenosis. It is possible that among those who were transferred shortly after extubation and lost for follow-up, some developed stridor in a later stage. Also, the presence of gastro-esophageal reflux has been associated with post-extubation injuries like the formation of granulomas, but there is debate regarding the value of medical management [18, 30]. Although a high level of evidence is lacking, the management of reflux might be beneficial. We did not study the presence or treatment of gastro-esophageal reflux in our study.

We think that despite the limitations of this study, we have convincingly shown that there is a high incidence of post-extubation stridor, which generally reacts on conservative treatment and the incidence of a laryngotracheal stenosis is low. Young age, intubation on the scene, and the use of (old-fashioned) cuffed tubes are contributing factors to post-extubation stridor. This offers clinicians the possibility to anticipate on difficult situation and to minimize the risk of developing laryngeal impairment. Clinicians should have extra awareness for post-extubation laryngeal lesions in young trauma patients intubated at the scene with a cuffed tube and they can anticipate on the situation, for example, by administering corticosteroids before extubation.

## Conclusion

This study reveals a high incidence of post-extubation stridor at a tertiary referral PICU, but a low incidence of laryngeal injury necessitating surgical intervention. Young age, intubation on the scene, and the use of cuffed tubes were associated with a significant increased risk of post-extubation stridor. Awareness of these factors gives the possibility to anticipate on the situation and to minimize laryngeal injury and their consequences.

## References

[1] Duynstee ML, de Krijger RR, Monnier P, Verwoerd CD, Verwoerd-Verhoef HL (2002). Subglottic stenosis after endolaryngeal intubation in infants and children: result of wound healing processes. Int J Pediatr Otorhinolaryngol.

[2] Holzki J, Laschat M, Puder C (2009). Iatrogenic damage to the pediatric airway mechanisms and scar development. Paediatr Anaesth.

[3] Bharti B, Syed KA, Ebenezer K, Varghese AM, Kurien M (2016). Post intubation laryngeal injuries in a pediatric intensive care unit of tertiary hospital in India: a fibreoptic endoscopic study. Int J Pediatr Otorhinolaryngol.

[4] de Lima ES, de Oliveira MA, Barone CR, Dias KM, de Rossi SD, Schweiger C (2016). Incidence and endoscopic characteristics of acute laryngeal lesions in children undergoing endotracheal intubation. Braz J Otorhinolaryngol.

[5] Manica D, de Souza Saleh Netto C, Schweiger C, Sekine L, Eneas LV, Pereira DR (2017). Association of endotracheal tube repositioning and acute laryngeal lesions during mechanical ventilation in children. Eur Arch Otorhinolaryngol..

[6] Schweiger C, Marostica PJ, Smith MM, Manica D, Carvalho PR, Kuhl G (2013). Incidence of post-intubation subglottic stenosis in children: prospective study. J Laryngol Otol.

[7] Smith MM, Kuhl G, Carvalho PR, Marostica PJ (2007). Flexible fiber-optic laryngoscopy in the first hours after extubation for the evaluation of laryngeal lesions due to intubation in the pediatric intensive care unit. Int J Pediatr Otorhinolaryngol.

[8] Gomes Cordeiro AM, Fernandes JC, Troster EJ (2004). Possible risk factors associated with moderate or severe airway injuries in children who underwent endotracheal intubation. Pediatr Crit Care Med.

[9] Jorgensen J, Wei JL, Sykes KJ, Klem SA, Weatherly RA, Bruegger DE (2007). Incidence of and risk factors for airway complications following endotracheal intubation for bronchiolitis. Otolaryngol Head Neck Surg.

[10] Manica D, Schweiger C, Marostica PJ, Kuhl G, Carvalho PR (2013). Association between length of intubation and subglottic stenosis in children. Laryngoscope.

[11] Nascimento MS, Prado C, Troster EJ, Valerio N, Alith MB, Almeida JF (2015). Risk factors for post-extubation stridor in children: the role of orotracheal cannula. Einstein.

[12] Sherman JM, Lowitt S, Stephenson C, Ironson G (1986). Factors influencing acquired subglottic stenosis in infants. J Pediatr.

[13] Suzumura H, Nitta A, Tanaka G, Kuwashima S, Hirabayashi H (2000). Role of infection in the development of acquired subglottic stenosis in neonates with prolonged intubation. Pediatr Int.

[14] Albert DM, Mills RP, Fysh J, Gamsu H, Thomas JN (1990). Endoscopic examination of the neonatal larynx at extubation: a prospective study of variables associated with laryngeal damage. Int J Pediatr Otorhinolaryngol.

[15] Dankle SK, Schuller DE, McClead RE (1986). Risk factors for neonatal acquired subglottic stenosis. Ann Otol Rhinol Laryngol.

[16] Thomas RE, Rao SC, Minutillo C, Hullett B, Bulsara MK (2018). Cuffed endotracheal tubes in infants less than 3 kg: a retrospective cohort study. Paediatr Anaesth.

[17] Zawadzka-Głos L, Obarska A (2015). Effect of duration of intubation on post intubation laryngeal changes in children. New Med..

[18] Jang M, Basa K, Levi J (2018). Risk factors for laryngeal trauma and granuloma formation in pediatric intubations. Int J Pediatr Otorhinolaryngol.

[19] Shi F, Xiao Y, Xiong W, Zhou Q, Huang X (2016). Cuffed versus uncuffed endotracheal tubes in children: a meta-analysis. J Anesth.

[20] Thomas R, Rao S, Minutillo C (2016). Cuffed endotracheal tubes for neonates and young infants: a comprehensive review. Arch Dis Child Fetal Neonatal Ed.

[21] Weiss M, Dullenkopf A, Fischer JE, Keller C, Gerber AC, European Paediatric Endotracheal Intubation Study G (2009). Prospective randomized controlled multi-centre trial of cuffed or uncuffed endotracheal tubes in small children. Br J Anaesth..

[22] da Silva O, Stevens D (1999). Complications of airway management in very-low-birth-weight infants. Biol Neonate.

[23] Deakers TW, Reynolds G, Stretton M, Newth CJ (1994). Cuffed endotracheal tubes in pediatric intensive care. J Pediatr.

[24] Schweiger C, Eneas LV, Manica D, Netto CSS, Carvalho PRA, Piva JP (2020). Accuracy of stridor-based diagnosis of post-intubation subglottic stenosis in pediatric patients. J Pediatr (Rio J).

[25] Ehrlich PF, Seidman PS, Atallah O, Haque A, Helmkamp J (2004). Endotracheal intubations in rural pediatric trauma patients. J Pediatr Surg.

[26] Ashtekar CS, Wardhaugh A (2005). Do cuffed endotracheal tubes increase the risk of airway mucosal injury and post-extubation stridor in children?. Arch Dis Child.

[27] De Orange FA, Andrade RG, Lemos A, Borges PS, Figueiroa JN, Kovatsis PG (2017). Cuffed versus uncuffed endotracheal tubes for general anaesthesia in children aged eight years and under. Cochrane Database Syst Rev..

[28] Khemani RG, Randolph A, Markovitz B (2009). Corticosteroids for the prevention and treatment of post-extubation stridor in neonates, children and adults. Cochrane Database Syst Rev..

[29] Veldhoen ES, Smulders CA, Kappen TH, Calis JC, van Woensel J, Raymakers-Janssen PA (2017). Post-extubation stridor in Respiratory Syncytial Virus bronchiolitis: is there a role for prophylactic dexamethasone?. PLoS ONE.

[30] Carter J, Rahbar R, Brigger M, Chan K, Cheng A, Daniel SJ (2016). International Pediatric ORL Group (IPOG) laryngomalacia consensus recommendations. Int J Pediatr Otorhinolaryngol.

